# Heterogeneity of Diffusion-Weighted Imaging in Tumours and the Surrounding Stroma for Prediction of Ki-67 Proliferation Status in Breast Cancer

**DOI:** 10.1038/s41598-017-03122-z

**Published:** 2017-06-06

**Authors:** Ming Fan, Ting He, Peng Zhang, Juan Zhang, Lihua Li

**Affiliations:** 1 Institute of Biomedical Engineering and Instrumentation, Hangzhou Dianzi University, Hangzhou, China; 2 Department of Radiology, Zhejiang Cancer Hospital, Zhejiang, Hangzhou, 310010 China

## Abstract

Breast tissue heterogeneity is related to risk factors that lead to more aggressive tumour growth and worse prognosis, yet such heterogeneity has not been well characterized. The aim of this study is to reveal the heterogeneous signal patterns of the apparent diffusion coefficient (ADC) of a tumour and its surrounding stromal tissue and to predict the Ki-67 proliferation status in oestrogen receptor (ER)-positive breast cancer patients. A dataset of 82 patients who underwent diffusion-weighted imaging (DWI) examination was collected. The ADC map was segmented into regions comprising the tumour and the surrounding stromal shells. To reflect correlations between each region in terms of its mean ADC value, a functional graph was constructed consisting of nodes as regions and edges as interactions between two nodes. Analysis of the graph revealed a higher average degree in samples over-expressing Ki-67 than in samples with low Ki-67 expression. In the low-Ki-67 group, most of the identified edges represented correlations between adjacent regions, whereas additional edges representing correlations between non-adjacent regions were found in the high-Ki-67 group. The ADC signal in various breast stromal regions surrounding the tumour showed a discriminative pattern and would be valuable for estimating the Ki-67 proliferation status by DWI.

## Introduction

Breast cancer is known to be a heterogeneous disease, and the different subtypes can be defined by the immunohistochemical (IHC) approach based on oestrogen receptor (ER), progesterone receptor (PR), and human epidermal growth factor receptor 2 (HER2) and Ki-67 expression levels. Approximately 70% of human breast cancer tumours are ER positive^1^, with a generally favourable prognosis, though a subset will experience relapse. In ER-positive breast cancer, the Ki-67 index, which is a proliferation marker, has been used to distinguish luminal B from luminal A cancer^2^. Luminal B tumours are more often high grade and have higher Ki-67 index than luminal A tumours. Thus, patients with luminal B breast cancer often have worse prognosis than patients with luminal A breast cancer^3, 4^. Therefore, it is of paramount importance to identify this subgroup of ER-positive patients with a relatively poor prognosis who may benefit from adjuvant chemotherapy.

A large population-based cohort study identified Ki-67 as an independent prognostic parameter for disease-free survival and overall survival^5^. Recent data suggest that patients with a lower Ki-67 level more often undergo pathological complete response (pCR)^6–8^ and a high Ki-67 level (above 14%) defines a high-risk group in terms of prognosis^9^. Moreover, the Ki-67 proliferation index reflects the extent of proliferative activity, an indicator of tumour aggressiveness^10^, and is a reliable identifier of more aggressive growth in breast cancer^9^. During growth, a tumour constantly interacts with the surrounding microenvironment by releasing extracellular signals, thereby promoting increased angiogenesis and microvessel density and leading to a tumour-progression-related microenvironment^11, 12^. The altered tumour microenvironment can in turn contribute to remodelling of the extracellular matrix (ECM), which changes stromal properties by altering matrix cross-linking, increasing collagen deposition and reorganizing fibres and consequently leads to a more rigid tumour-associated stroma^13^. Therefore, the tumour microenvironment is spatially heterogeneous, and the patterns and correlations between a tumour and its surrounding stromal tissue are contributed to the characterization of intrinsic features of breast cancer.

Diffusion-weighted imaging (DWI) is an approach that differs from conventional magnetic resonance imaging (MRI) techniques in that it measures the mobility of water within tissues, providing information about tissue cellularity and cell membranes integrity^14^, and it is sensitive to changes in water diffusion in the intracellular and extracellular spaces^13^. As such, flow-insensitive apparent diffusion coefficients (ADCs) may provide a more accurate estimation of the cellularity of the tumour microenvironment by minimizing any vascular contribution^15^. Compared with benign tumours, lower ADC values, which reflect restrictions in water diffusion, are observed in malignant breast lesions^16, 17^, and studies have identified that ADC values can be helpful for predicting response to neoadjuvant chemotherapy in breast cancer^18, 19^. Related studies have reported a correlation between ADC values and pathologic factors^20–24^ as well as biomarkers such as tumour cellularity and Ki-67 expression levels in luminal-type breast cancer^25^, mucinous breast cancer^26^ and ER-positive breast cancer^27, 28^. Conversely, another study found no association between ADC values and prognostic factors^29^.

Although previous studies have examined associations between stromal features surrounding the tumour region^30, 31^ and the pathological status, to our knowledge, no study has investigated to analyse the heterogeneity of ADC patterns in stromal regions. In addition, no predictive models have been applied to differentiate Ki-67 status using these features within a given cohort. Here, we investigate features based on DWI in the peritumoural stroma region to evaluate this prognosis biomarker in breast cancer. Our approach is different from the work presented in previous studies^30, 31^, in which statistical features were extracted based on the tumour and its surrounding stroma. In addition, we also examine correlations of mean ADC values between each peritumour stroma, which could help provide possible information for the differentiation of stromal connections between aggressive and non-aggressive cancers.

In this study, we analysed the heterogeneity of tumour and peritumoural sub-regions of breast stroma on DWI and evaluated the ability of MR features using a multivariate logistic classifier to distinguish ER positive breast cancers with low Ki-67 levels from those with high Ki-67 levels.

## Methods

### Clinical Demographics

This is a retrospective study approved by the Internal Research Review and Ethical Committee of the Zhejiang Cancer Hospital and informed consent was obtained from all patients. All methods were carried out in accordance with the relevant guidelines. We collected an initial dataset of 183 patients at Zhejiang Cancer Hospital, China. Of the total, 52 patients with no pathologic examination or incomplete pathology data were excluded. Additionally, 9 patients who underwent breast cancer treatment (e.g., chemotherapy or radiation therapy) prior to MRI were also eliminated from the cohort, and 40 ER-negative patients were removed from the dataset. Ultimately, 82 patients who met the selection criteria were included in our dataset for analysis.

Table 1 summarizes tumour and patient characteristics by Ki-67 proliferation status. Ki-67 >14% was considered high expression and Ki-67 <14% was considered low expression. The mean age of the patients was 51.65 years (range: 21–71 years), and all were Han Chinese. In this sample, 5 of the subjects were premenopausal and 65 postmenopausal; 12 were of unknown status. The distribution of invasive breast cancers by tumour type was as follows: 57 invasive ductal carcinomas, 3 lobular carcinomas, and 22 poorly differentiated adenocarcinomas. In the cohort, 54 (65.9%) cases showed Ki-67 over-expression and 28 (34.1%) low Ki-67 expression. There was no significant difference in receptor status or tumour volume for patients with high vs low Ki-67 expression.

**Table 1:** Tumour characteristics.

Characteristic	All	High >= 14%	Low < 14%	P-value
	82	(n = 54) (%)	(n = 28) (%)	
PR^a^				0.527
Positive	69	44 (54)	25 (30)	
Negative	13	10 (12)	3 (4)	
Histopathology^a^				0.818
Ductal	51	32 (39)	19 (23)	
Intraductal	5	4 (5)	1 (1.2)	
lobular	3	2 (2.4)	1 (1.2)	
Others	23	16 (20)	7 (7.3)	
Menopausal status^a^				0.064
Premenopausal	5	1 (1.2)	4 (5)	
Postmenopausal	65	46 (56)	19 (23)	
Others	12	7 (8.5)	5 (6)	
Age (year)^b^	51.65 (27–71)	50.41 (27–67)	54.04 (38–71)	0.100
Tumour Volume (mm^3^)^b^	12628	15846	7341	0.128
	(180–72000)	(1000–72000)	(180–31500)	

^a^Data were tested using the Fisher’s exact test.

^b^Data were tested using ANOVA.

### MR Image Acquisition

All patients were scanned in the prone position using an MRI scanner with magnetic field strength of 3.0 T (MAGNETOM Verio; Siemens Healthcare, Erlangen, Germany) and a dedicated eight-channel double-breast coil. A DWI sequence was obtained with the following parameters: field of view (FOV) 104 × 320 mm^2^; flip angle 90°; TR (Repetition Time ms) 7000; TE (Echo Time ms) 85 ms; slice thickness 6 mm; spacing between slices 6.6 mm; b values 50 and 1000 sec/mm^2^; acquisition matrix 220 × 72; pixel bandwidth 1196 Hz per pixel; and in-plane resolution 1.45 × 1.45 mm^2^.

### DW MR Imaging Analysis

**Figure 1:**
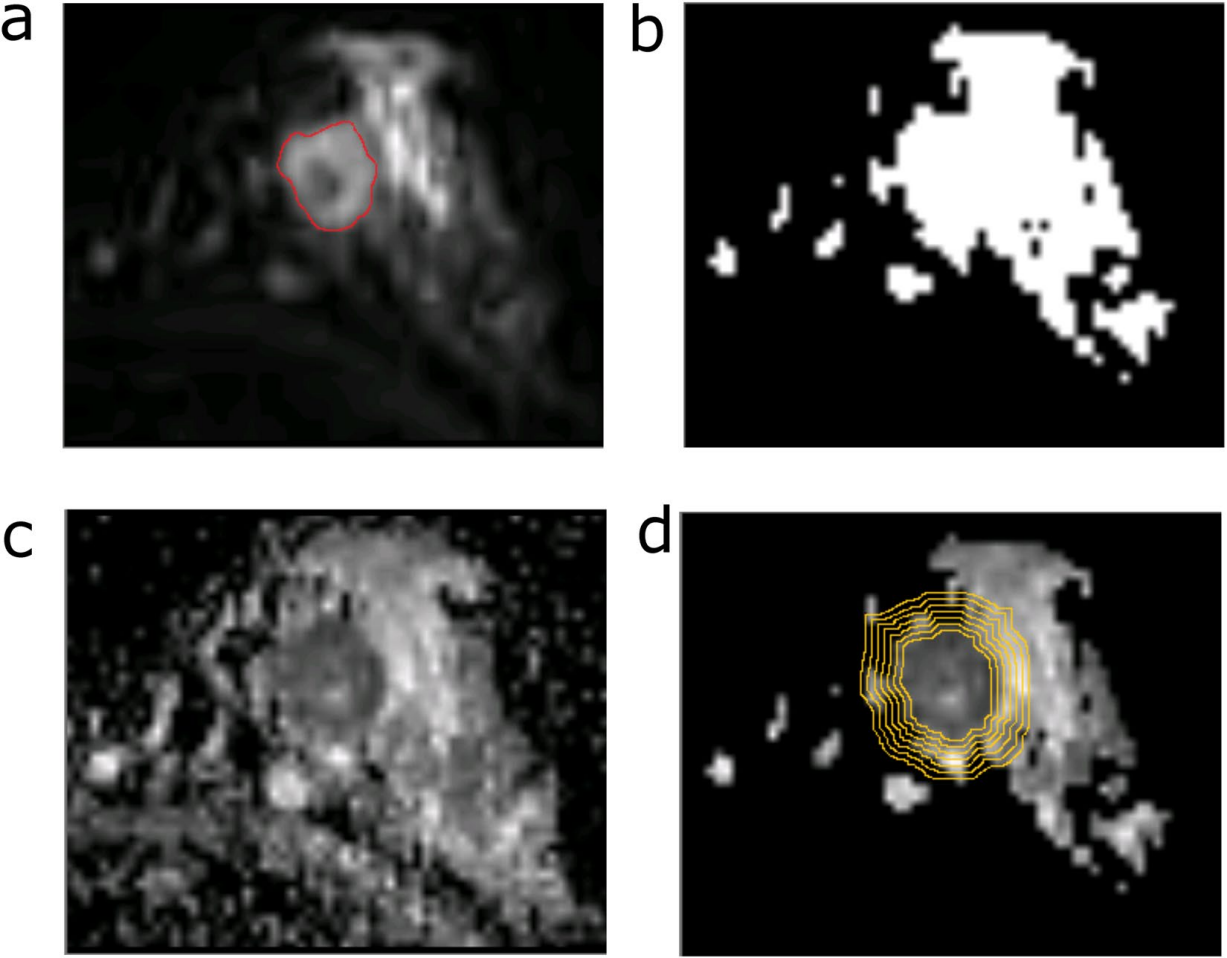
An example of a peritumoural stromal map in the left breast of a patient. (**a**) Diffusion-weighted image at b = 50 s/mm^2^ shows a mass with high signal intensity (red circle). (**b**) Fibroglandular tissue, excluding the fat from the breast, was segmented on the DWI b = 50 image. (**c**) ADC image of the breast. (**d**) The fibroglandular proximity map was applied to an ADC image to calculate the proximal peritumoural stromal ADC map.

DWI ADC maps were calculated on a pixel-by-pixel basis according to the equation ADC = −ln[S_1_/S_0_]/(b_1_ − b_0_), where S_0_ and S_1_ are the signal intensities in the image obtained by two gradient factors, b_1_ and b_0_ (b_0_ = 50 s/mm^2^ and b_1_ = 1000 s/mm^2^, respectively). To standardize the image analysis as much as possible, a representative ADC map that shows the largest dimension of the tumour was selected. Fibroglandular tissue was segmented on the DWI b = 50 image, excluding fat from the breast, based on a fuzzy C-means clustering procedure (Fig. 1). The tumour regions of interests (ROIs) were manually drawn to the margin of the whole tumour with high signal intensity on DWI at b = 50 and then aligned to the ADC map. To assess its heterogeneity in terms of ADC values, the whole tumour was included for analysis. The ADC images were manually segmented into the inner tumour, tumour boundary, peritumoural region, and more distant region from the tumour according to the manually drawn tumour ROIs based on DWI.

### DWI Feature Extraction

#### Histogram features in peritumour stromal regions

In our study, the resolution of DWI was 1.45 × 1.45 mm, and we selected stromal shell, with a 4 pixel width (1.45 mm × 4 pixel = 5.8 mm). We defined six regions similar to the procedure by Sin *et al*.^31^. These regions are as follows: (1) an inner section of the tumour at least 2 pixels inside the tumour edge, i.e., *S*_I_; (2) the whole tumour (*S*_T_); (3) the boundary region of the tumour, from −2 to 2 pixels of the tumour edge (*S*_b_); (4) peritumour stromal shell, with a 4 pixel width next to the tumour boundary, i.e., *S*_P_; (5) the intermediate stromal shell, with a 4 pixel width neighbouring the proximal stromal region (*S*_M_); and (6) the distant stromal shell (*S*_D_), with a 4 pixel width outside *S*_M_. For each region, eight histogram statistical features were calculated: mean, min, variance, mean, interquartile range, range, skewness, and kurtosis of ADC values.

#### Ratio between breast subregions

We also calculated the ratio of statistical measurements between each of the two regions including the tumour and its surrounding stroma. For example, the ratio of the interquartile ranges of the tumour boundary and peritumoural proximal stromal regions, i.e., *S*_B_ and *S*_P_, was calculated.

### Pathologic Assessment

Immunohistochemical staining was performed using streptavidin-peroxidase. The pathology report from the initial breast biopsy or surgery was used to record the ER, PR, and HER2 statuses of each invasive cancer. Samples were scored as positive for ER or PR by immunohistochemistry (IHC) when at least 1% of the tumour cell nuclei showed staining for ER or PR, respectively^32^. A sample was scored as HER2 positive following the American Society of Clinical Oncology (ASCO)/College of American Pathologists (CAP) guideline recommendations for HER2 testing in breast cancer^33^. A Ki-67 level greater than 14% was considered positive, and a level below 14% was considered negative.

### Statistical Analysis

Differences in tumour characteristics were assessed using a χ^2^ test or Fisher’s exact test when the expected value in any cell was less than five. Analysis of variance (ANOVA) was performed for comparison of ADC values between the high- and low-Ki-67 groups. A univariate logistic-based classifier was used to evaluate the performance of the individual features in differentiating high and low Ki-67 proliferation statuses. A multivariate logistic regression model was trained and validated to classify Ki-67 status using combined features. To evaluate the performance of the classifier, receiver-operating characteristic (ROC) analysis was performed, and the area under the ROC curve (AUC) was computed. Sensitivity and specificity were calculated using a Youden index determining the value that would maximize the average sensitivity and specificity^34^.

A functional breast stromal region association network, i.e., an undirected unweighted graph, was built with tumour or stromal regions as nodes and associations as edges. More specifically, Pearson’s correlation coefficient (PCC) of mean ADC values between two nodes were calculated, and edges/associations were established if Bonferroni-corrected P values of PCC were less than 0.05. Therefore, a network with 6 nodes was generated representing similar tissue characteristics such as cellularity and water content.

An evolutionary algorithm (EA)-based optimization method was applied to search for the optimal feature subset for classification. The mutation probability and crossover probability were set at 0.01 and 0.6, respectively. The EA chromosome population in each generation was 500, and the maximum number of generations was 200. The EA chromosome that achieved the highest AUC was selected to establish the optimal feature pool and build the optimal classifier. All statistical tests were two-tailed, and significance was set at a P value < 0.05. Statistical analyses were performed using Matlab R2013b (8.2).

To avoid overfitting of classifiers to the available data, the leave-one-out cross-validation (LOOCV) test was employed. Specifically, at each iteration of the LOOCV process, one sample was used for testing and the other for training. This procedure was repeated for each sample. In each evaluation, we performed a Wrapper Subset Evaluator (WSE) feature selection using all training cases to search for optimal features from the optimal feature subset pool generated from EA. Using these features, a multiclass logistic classifier was trained based on the training set and was tested on the one independent test case omitted. The importance of the predictive imaging features in the classifier was evaluated by selection frequencies of features over all LOOCV loops.

## Results

**Figure 2:**
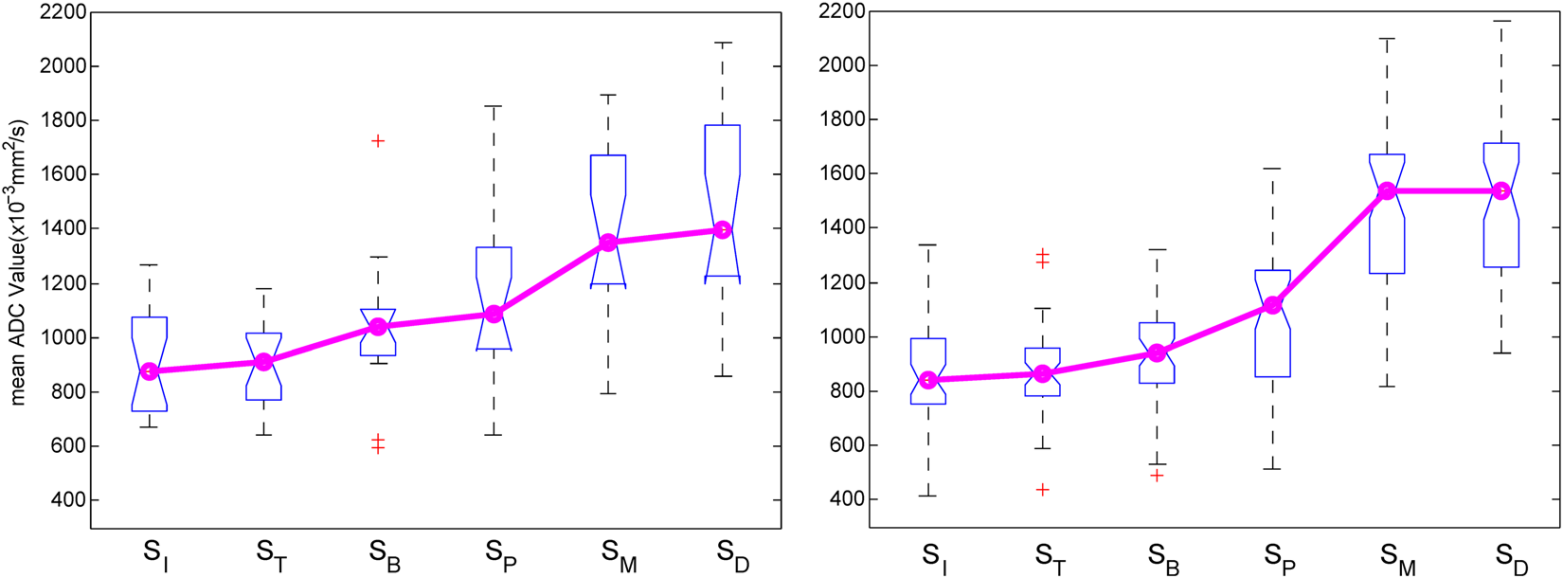
Comparison of median ADC values in six regions. An ascending trend (shown by the red trend line) for the ADC value was observed with (**a**) low Ki-67 proliferation status and (**b**) high Ki-67 proliferation status.

**Figure 3:**
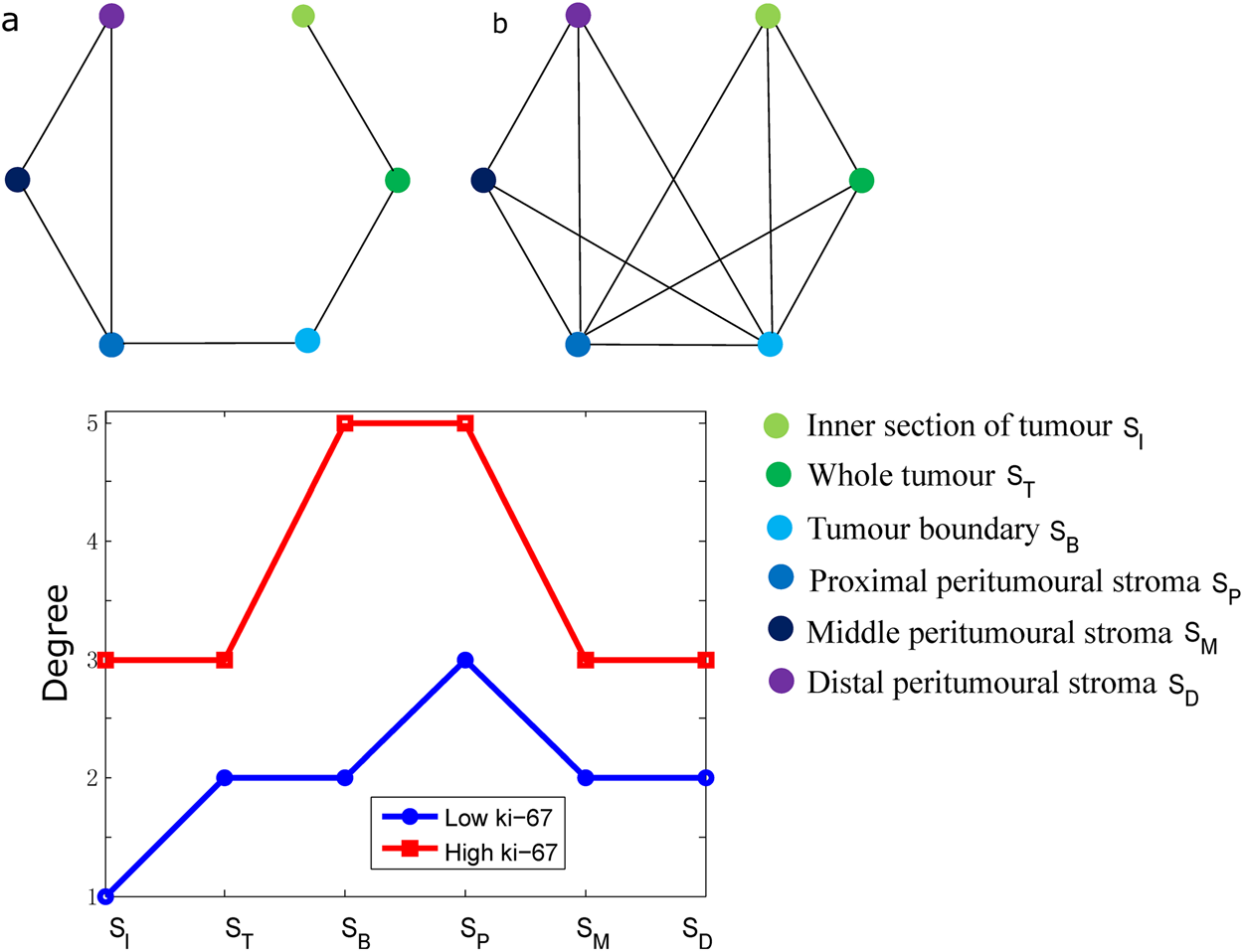
Connections between regions of tumours and peritumoural stroma. Graphs are shown for (**a**) low-Ki-67 and (**b**) high-Ki-67 groups. The degree for each region in the Ki-67 groups is shown in (**c**). Each node on the graph represents a tumour or peritumoural stroma, i.e., (1) inner section of a tumour, (2) whole tumour, (3) tumour boundary, (4) peritumoural stroma, (5) middle peritumoural stroma, and (6) distal peritumoural stroma. Each edge in this graph is defined by a significant correlation of mean ADC values between two regions (Bonferroni-corrected P value of Pearson’s correlation coefficient less than 0.05).

**Figure 4:**
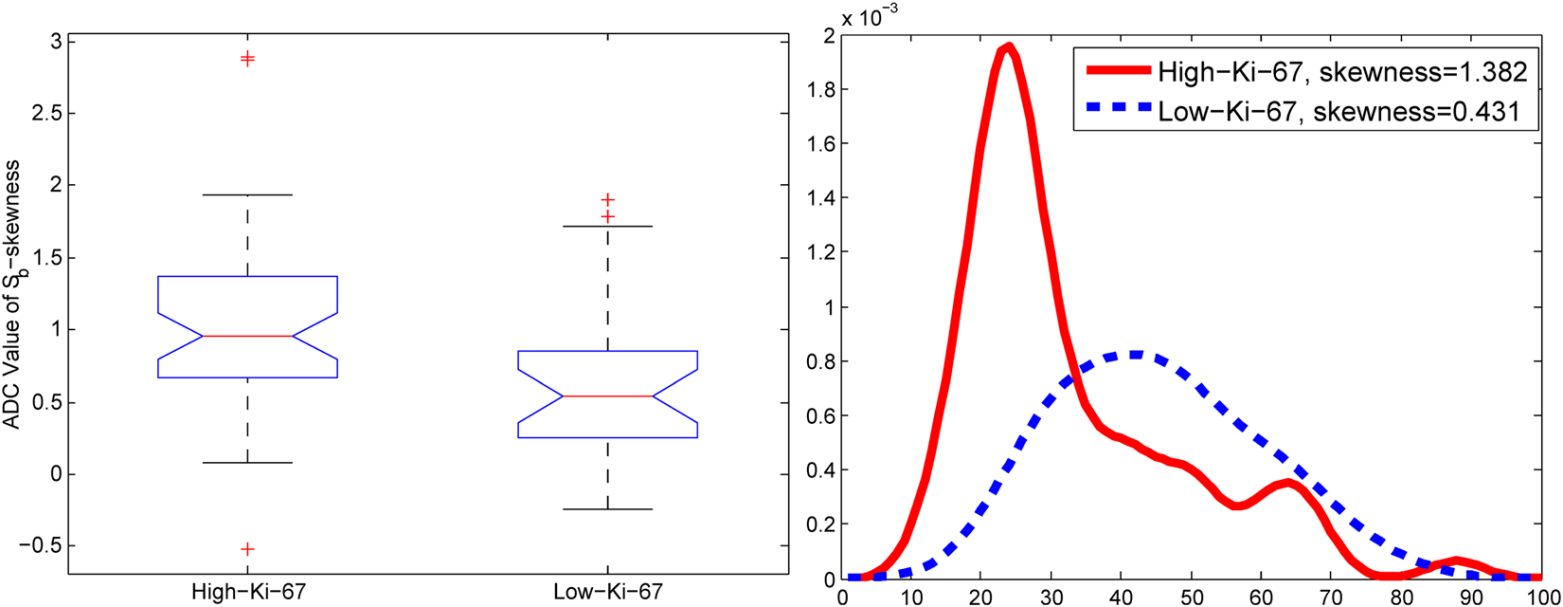
An example of the skewness feature of ADC values in the tumour boundary region. (**a**) Boxplot of skewness of ADC values in a tumour boundary for high and low Ki-67. (**b**) An example of high and low Ki-67 cases, with a density of ADC values in the tumour boundary.

In our study, we compared tumour mean ADC values according to histopathological features, as shown in Table 2. Furthermore, patterns of the ADC signal in the tumour and stromal regions of breast cancer were examined (Table S1 and Fig. 2). Exploratory analyses of a functional graph were established to investigate correlations of stromal regions in terms of mean ADC values (Fig. 3, Supplementary Fig. 1). Finally, we calculated 48 statistical features and 120 ratios of ADC features between these regions for classification of Ki-67 proliferation status (Fig. 4, Supplementary Figs 2 and 3, Table 3 and Supplementary Tables S2–S4).

**Table 2:** Comparison of ADC values in tumours according to histopathological features.

Characteristic	Number	Mean-ADC	P value
PR expression			0.804
Positive	69	0.888 ± 0.159	
Negative	13	0.875 ± 0.152	
Histology			0.643
Ductal	51	0.889 ± 0.175	
Intraductal	5	0.966 ± 0.139	
Lobular	3	0.893 ± 0.093	
Others	23	0.839 ± 0.127	
Menopausal status			0.052
Premenopausal	5	0.977 ± 0.102	
Postmenopausal	65	0.862 ± 0.162	
Others	12	0.963 ± 0.121

**Table 3:** Performance of prediction model using statistical features and ratio features.

Feature class	AUC	ACC	Sensitivity	Specificity
Regions				
S_I_	0.669 (0.539–0.801)	0.649	0.574	0.778
S_T_	0.620 (0.483–0.750)	0.622	0.617	0.630
S_B_	0.763 (0.649–0.873)	0.689	0.638	0.852
S_P_	0.721 (0.600–0.842)	0.668	0.915	0.544
S_M_	0.612 (0.466–0.758)	0.608	0.894	0.407
S_P_	0.609 (0.461–0.741)	0.635	0.766	0.444
Statistic features	0.868 (0.783–0.953)	0.839	0.851	0.815
Ratio features	0.912 (0.855–0.987)	0.879	0.809	0.915
All	0.933 (0862–0.997)	0.917	0.951	0.872

### Analysis of ADC patterns in tumour and peritumoural stromal regions

#### Comparison of tumour ADC values according to histopathological features

We first conducted comparison analyses of the mean ADC value within each subgroup in the whole tumour according to histological type, tumour diameter, tumour grade, and PR expression, as shown in Table 2. However, ANOVA showed no significant association for ADC values among histological groups.

#### Comparison of ADC values in peritumoural stromal regions

In addition to examination of ADC values in breast tumours according to histopathological characteristics, further analysis was conducted to investigate ADC signal patterns in the tumour and peritumoural stroma. As shown in Table 3, the whole tumour and inner tumour had the lowest ADC values among all six regions. In contrast, the middle and distal peritumoural stroma exhibited a higher level of ADC, a value close to that of normal tissue^35^. ANOVA revealed significant differences in mean ADC values among peritumoural stroma regions between the low and high Ki-67 proliferation statuses (p < 0.0001). An ascending order of median ADC values in the six regions is shown in Fig. 2. A significant increase in ADC values (P < 0.05) was observed between proximal and middle peritumoural stromal regions.

#### Correlation of ADC values in tumours and surrounding stromal regions

As a tumour spreads its characteristics into breast tissue, we assumed that there should be similar patterns of ADC values between a tumour and its surrounding stroma. Therefore, exploratory analyses were conducted to evaluate correlations among these regions. A graph was thus established with nodes as tumour/stromal regions and edges as significant (corrected P < 0.05 for PCC) correlations of mean ADC values between two nodes, as displayed in Fig. 3. It is interesting that higher total degrees were observed in samples over-expressing Ki-67 than in samples with low Ki-67 expression (Fig. 3(c)). The node representing the proximal peritumour stromal region (*S*_P_) had the highest degree, whereas that representing the inner tumour region had the lowest degree. Additionally, most edges in the low-Ki-67 group were found between nodes representing adjacent regions (e.g., *S*_I_ and *S*_T_; *S*_T_ and *S*_B_), though more edges representing correlations of non-adjacent regions were found in the high-Ki-67 group. These results suggest that the pattern for a tumour spreading to its surrounding region differed between breast cancers with greater versus lower proliferation. Supplementary Fig. 1 also shows a representative example of graphs with low and high total degrees for low- and high-Ki-67 cases, respectively.

### Classification experiments

#### DWI individual features for discriminating Ki-67 expression levels

Based on the above observations that ADC values and correlations among stromal regions differ between high- and low-Ki-67 groups, histogram features of ADC values in each stromal region and ratio features were further evaluated to discriminate Ki-67 status. We expected that different regions may have different performances in the classification of the Ki-67 expression level. The best two individual histogram features and ratio features in each stromal region, based on the AUC, accuracy, sensitivity and specificity, are provided in Table S2. The skewness of the ADC value in the tumour boundary was the best individual feature, with an AUC of 0.716 and 95% confidence intervals (CIs) from 0.583 to 0.845, a sensitivity of 0.723 and a specificity of 0.704. Higher skewness of the ADC value in the tumour boundary was associated with a high level of Ki-67 expression (Fig. 4(a)). Figure 4(b) also shows an example of a distribution of ADC values in the tumour boundary exhibiting high skewness in a high-Ki-67 tumour and low skewness, close to zero, with a low Ki-67 proliferation status. The two best ratio features for discriminating Ki-67 proliferation status were those of the means and interquartile ranges of proximal and middle peritumour stromal regions, which generated an AUC value of 0.659, with 95% CIs of 0.527 to 0.792, and an AUC value of 0.657, with 95% CIs of 0.579 to 0.786, respectively.

#### Combined features for classification of Ki-67 proliferation status

Further classification analysis using combined ADC features was performed for classifying Ki-67 proliferation status utilizing a multivariate logistic regression analysis model. To assess whether various stromal regions could have different performance, the classification models were applied in each region separately. Feature selection was performed for each model to produce the best subset of features for Ki-67 status discrimination. The performance of the classification models using features in the six proximal regions is illustrated in Table 3. The tumour boundary region achieved the best performance in terms of an AUC value of 0.763, with CIs of 0.649 to 0.873, whereas the proximal peritumour stromal region showed the second-best performance, with an AUC value of 0.716 and 95% CIs of 0.592 to 0.840.

The statistical features in each peritumoural stromal region were then combined to enhance the performance of the prediction model. A multivariate logistic regression model generated an AUC value of 0.868 using 10 statistical features from all regions (Table 3 and Supplementary Table S3). Supplementary Fig. S2 shows ROC curves of classifiers applying the statistical features in each of the six regions to classify Ki-67 proliferation status. In addition, the ratios of features between each pair of regions were also combined and entered into the prediction model. After feature selection, the classifier with 13 features yielded an AUC of 0.912, with 95% CIs of 0.855 to 0.987 (Table 3 and Supplementary Table S3). Comparisons of the prediction model in terms of ROCs using statistical features, ratio features and the combination of all features are displayed in Supplementary Fig. S3. The results indicated that the overall performance for ratio features was better than that of statistical features in the six regions. Detailed descriptions of the selected best subset of features are provided in Table S3. Finally, the multivariate classifier combining all features improved the classifier performance to an AUC of 0.933, which are significantly better than that of features in S_I_, S_T_, S_M_ and S_P_ (Supplementary Fig. S3 and Table 3).

Analysis of the contribution (importance) of individual features to the classification was based on the frequency of each feature selected in LOOCV loops, as shown in Table S4. The top 4 features with more than 30% frequencies in the prediction model were 2 ratio features and 2 statistical features. These features included the ADC skewness in the *S*_p_, the ratio of interquartile range ADC between *S*_I_ and *S*_T_, the ADC skewness in the tumour boundary and, the ratio of minimum ADC value between *S*_I_ and *S*_P_. Among these, most (10 out of 15) are ratio features, indicating that the ratios of features between distinct regions contribute more to the classification model than statistical features.

## Discussion

In this study, we analysed patterns of ADC values in tumour and peritumoural stroma, and a predictive model was built to evaluate the performance of applying imaging features to predict Ki-67 proliferation status in patients with ER-positive invasive breast cancer. The tumour and its proximal regions were identified, and correlations between each region in terms of ADC values were established. A distinct pattern of connections between high- and low-Ki-67 proliferation status samples in the tumour and peritumour stroma was found. Further experiments showed that statistical features in the tumour boundary as well as in the proximal peritumoural stroma have higher predictive power than the other regions.

In the present study, ADC values were grouped in ascending order in breast stromal regions when the distance from the tumour edge was increased, which is in line with previous studies^13, 31^. Additionally, the features in the tumour boundary and the proximal peritumoural stroma showed higher performance in discriminating expression of Ki-67 compared to those in other regions. This result is in agreement with a recent study that identified more significant associations with Ki-67 status in the tumour boundary and proximal peritumoural stroma^31^. The results of our study indicate that the breast stromal band proximal to the tumour is important for the analysis of tumour characteristics.

Based on graph analysis, high total degrees representing correlations between stromal regions were observed for the high-Ki-67 expression group than the low-Ki-67 group. Moreover, higher degrees were observed for nodes representing the tumour boundary and proximal peritumoural stroma. The results suggest that compared to a low-Ki-67 tumour, a high-Ki-67 tumour, with more proliferation, may spread its characteristics to a greater distance in the proximal surrounding regions. Additionally, normal-appearing stroma surrounding a tumour would exhibit more abnormities in samples highly expressing Ki-67. This finding of our study is consistent with the fact that high-Ki-67 cases are more aggressive with regard to tumour cell proliferation than low-Ki-67 cases. Because the features of ADC values reflect the Ki-67 status, measuring patterns of ADC values may facilitate an understanding of patients’ clinical presentation. Therefore, DWI is a promising unenhanced tool for detecting differences in water mobility that reflect the tissue microenvironment.

In our study, the two best individual features for predicting of Ki-67 status were the skewness and interquartile range on histogram analysis, which are regarded as a reflection of tumour heterogeneity^4^. The possible explanation for this finding is that a high-Ki-67 tumour, with characteristics of more aggressive growth, might have a more heterogonous tumour microenvironment affecting its surrounding proximal stromal regions. Furthermore, the results of our study suggest that histogram analysis of pixel-based data in addition to mean ADC values could provide more predictive information in ER-positive breast cancer than conventional features such as the mean ADC value^8^.

Our study has several limitations. First, this is a retrospective study with limited statistical power due to the relatively small sample size. Thus, our study results must be confirmed in future studies with larger datasets. Second, this is a single-institution study without a validation cohort. As systematic fluctuations exist among different medical institutions caused by the use of different b value protocols for breast cancer patients, whether our image features of various peritumour stroma can be optimally applied to DW images acquired from other medical institutions must be tested in future studies. Third, only first-order histogram features were examined for prediction, whereas second-order spatial histogram features such as the textural features and the pathologic information, i.e., histologic grade and lymph node status, were not included in the model. Further analysis that uncovers more tumours and their surrounding stromal features should help to better understand the relationships between DWI and the prognosis of breast cancer.

In conclusion, our results suggest that patterns of ADC values in tumours and stromal regions may be predictive of tumour aggressiveness indicators, i.e., Ki-67, in ER-positive patients. Statistical features of stromal regions according to the distance of the tumour boundary were correlated with tumour classification, which could provide additional information about tumour heterogeneity in such cases. This is the first exploratory study examining features of ADC values in tumours and stroma to reflect heterogeneous patterns in differentiation of Ki-67 proliferation status. A further prospective study with a large number of patients is needed to confirm our results.

## Electronic supplementary material

Supplementary Figure 1

## References

[1] Lumachi, F., Brunello, A., Maruzzo, M., Basso, U. & Basso, S. M. Treatment of estrogen receptor-positive breast cancer. *Current medicinal chemistry* **20**, 596–604 (2013). 10.2174/092986713804999303 23278394

[2] Sheri A, Dowsett M (2012). Developments in Ki67 and other biomarkers for treatment decision making in breast cancer. Annals of oncology: official journal of the European Society for Medical Oncology.

[3] Kontzoglou K (2013). Correlation between Ki67 and breast cancer prognosis. Oncology.

[4] Coates AS, Colleoni M, Goldhirsch A (2012). Is adjuvant chemotherapy useful for women with luminal a breast cancer?. Journal of clinical oncology: official journal of the American Society of Clinical Oncology.

[5] Inwald EC (2013). Ki-67 is a prognostic parameter in breast cancer patients: results of a large population-based cohort of a cancer registry. Breast cancer research and treatment.

[6] Kim KI (2014). Ki-67 as a predictor of response to neoadjuvant chemotherapy in breast cancer patients. Journal of breast cancer.

[7] Fasching PA (2011). Ki67, chemotherapy response, and prognosis in breast cancer patients receiving neoadjuvant treatment. BMC cancer.

[8] Brown JR, DiGiovanna MP, Killelea B, Lannin DR, Rimm DL (2014). Quantitative assessment Ki-67 score for prediction of response to neoadjuvant chemotherapy in breast cancer. Laboratory investigation; a journal of technical methods and pathology.

[9] Yerushalmi R, Woods R, Ravdin PM, Hayes MM, Gelmon KA (2010). Ki67 in breast cancer: prognostic and predictive potential. The Lancet. Oncology.

[10] Gasparini, G. *et al*. Breast cancer cell kinetics: immunocytochemical determination of growth fractions by monoclonal antibody Ki-67 and correlation with flow cytometric S-phase and with some features of tumor aggressiveness. *Anticancer research* **11**, 2015–2021 (1991). 1776834

[11] Tlsty TD, Coussens LM (2006). Tumor stroma and regulation of cancer development. Annual review of pathology.

[12] Mao Y, Keller ET, Garfield DH, Shen K, Wang J (2013). Stromal cells in tumor microenvironment and breast cancer. Cancer metastasis reviews.

[13] Yili Z (2009). The value of diffusion-weighted imaging in assessing the ADC changes of tissues adjacent to breast carcinoma. BMC cancer.

[14] Hamstra DA, Rehemtulla A, Ross BD (2007). Diffusion magnetic resonance imaging: a biomarker for treatment response in oncology. Journal of clinical oncology: official journal of the American Society of Clinical Oncology.

[15] Koh DM, Collins DJ (2007). Diffusion-weighted MRI in the body: applications and challenges in oncology. AJR. American journal of roentgenology.

[16] El Khouli RH (2010). Diffusion-weighted Imaging Improves the Diagnostic Accuracy of Conventional 3.0-T Breast MR Imaging. Radiology.

[17] Cai, H. M., Peng, Y. X., Ou, C. W., Chen, M. S. & Li, L. Diagnosis of Breast Masses from Dynamic Contrast-Enhanced and Diffusion-Weighted MR: A Machine Learning Approach. *PloS one* **9**, doi:ARTN e87387 10.1371/journal.pone.0087387 (2014). 10.1371/journal.pone.0087387 PMC3909149 24498092

[18] Liu SG (2015). Diffusion-weighted imaging in assessing pathological response of tumor in breast cancer subtype to neoadjuvant chemotherapy. Journal of Magnetic Resonance Imaging.

[19] Park SH (2010). Diffusion-weighted MR Imaging: Pretreatment Prediction of Response to Neoadjuvant Chemotherapy in Patients with Breast Cancer. Radiology.

[20] Martincich L (2012). Correlations between diffusion-weighted imaging and breast cancer biomarkers. European radiology.

[21] Park SH, Choi HY, Hahn SY (2015). Correlations between apparent diffusion coefficient values of invasive ductal carcinoma and pathologic factors on diffusion-weighted MRI at 3.0 Tesla. Journal of magnetic resonance imaging: JMRI.

[22] Kim EJ (2015). Histogram Analysis of Apparent Diffusion Coefficient at 3.0 T: Correlation With Prognostic Factors and Subtypes of Invasive Ductal Carcinoma. Journal of Magnetic Resonance Imaging.

[23] Jones EF (2013). MRI enhancement in stromal tissue surrounding breast tumors: association with recurrence free survival following neoadjuvant chemotherapy. PloS one.

[24] Jeh SK (2011). Correlation of the apparent diffusion coefficient value and dynamic magnetic resonance imaging findings with prognostic factors in invasive ductal carcinoma. Journal of magnetic resonance imaging: JMRI.

[25] Mori N (2015). Luminal-type breast cancer: correlation of apparent diffusion coefficients with the Ki-67 labeling index. Radiology.

[26] Onishi N (2015). Apparent diffusion coefficient as a potential surrogate marker for Ki-67 index in mucinous breast carcinoma. Journal of magnetic resonance imaging: JMRI.

[27] Shin HJ (2016). Tumor apparent diffusion coefficient as an imaging biomarker to predict tumor aggressiveness in patients with estrogen-receptor-positive breast cancer. NMR in biomedicine.

[28] Shin, J. K. & Kim, J. Y. Dynamic contrast-enhanced and diffusion-weighted MRI of estrogen receptor-positive invasive breast cancers: Associations between quantitative MR parameters and Ki-67 proliferation status. *Journal of magnetic resonance imaging*: *JMRI*, doi:10.1002/jmri.25348 (2016). 10.1002/jmri.25348 27313102

[29] Kim SH (2009). Diffusion-weighted imaging of breast cancer: correlation of the apparent diffusion coefficient value with prognostic factors. Journal of magnetic resonance imaging: JMRI.

[30] McLaughlin RL (2014). High resolution *in vivo* characterization of apparent diffusion coefficient at the tumor-stromal boundary of breast carcinomas: a pilot study to assess treatment response using proximity-dependent diffusion-weighted imaging. Journal of magnetic resonance imaging: JMRI.

[31] Shin HJ (2016). Characterization of tumor and adjacent peritumoral stroma in patients with breast cancer using high-resolution diffusion-weighted imaging: Correlation with pathologic biomarkers. European journal of radiology.

[32] Hammond ME, Hayes DF, Wolff AC, Mangu PB, Temin S (2010). American society of clinical oncology/college of american pathologists guideline recommendations for immunohistochemical testing of estrogen and progesterone receptors in breast cancer. J Oncol Pract.

[33] Wolff AC (2013). Recommendations for human epidermal growth factor receptor 2 testing in breast cancer: American Society of Clinical Oncology/College of American Pathologists clinical practice guideline update. J Clin Oncol.

[34] Schisterman, E. F., Perkins, N. J., Liu, A. & Bondell, H. Optimal cut-point and its corresponding Youden Index to discriminate individuals using pooled blood samples. *Epidemiology* **16**, 73–81 (2005). 10.1097/01.ede.0000147512.81966.ba 15613948

[35] Bogner W (2009). Diffusion-weighted MR for Differentiation of Breast Lesions at 3.0 T: How Does Selection of Diffusion Protocols Affect Diagnosis?. Radiology.

