# Supplementary material for: Heterogeneity of Diffusion-Weighted Imaging in Tumours and the Surrounding Stroma for Prediction of Ki-67 Proliferation Status in Breast Cancer
Source: Sci Rep. 2017 Jun 6;7:2875. doi: 10.1038/s41598-017-03122-z (PMC5460128; doi:10.1038/s41598-017-03122-z)
Supplement: Supplementary file 1 — Supplementary Figure 1 [file 41598_2017_3122_MOESM1_ESM.doc]

**Supplementary Information**

**Heterogeneity of Diffusion-Weighted Imaging in the Tumour and Its Surrounding Stroma for Prediction of Ki-67 Proliferation Status in Breast Cancer**

Ming Fan^1§^, Ting He^1§^, Peng Zhang^1^, Juan Zhang^2^, Lihua Li^1^*

^1^Institute of Biomedical Engineering and Instrumentation, Hangzhou Dianzi University, Hangzhou, China

^2^Zhejiang Cancer Hospital, Zhejiang Hangzhou, China

^§^These authors contribute equally to this work

Corresponding to Lihua Li, Email: lilh@hdu.edu.cn

College of life information and instrument engineering, Hangzhou Dianzi University, Hangzhou, China, 310018

**Supplementary Tables**

**Supplementary Table S1. Comparison of mean ADC values between K**i-67 expression levels in six regions

|  |  | Low (p <0.0001) | High (p <0.0001) | P |
| --- | --- | --- | --- | --- |
|  | S_I_ | 0.901±0.182 | 0.873±0.179 | 0.623 |
|  | S_T_ | 0.905±0.154 | 0.846±0.156 | 0.288 |
|  | S_B_ | 1.043±0.241 | 0.947±0.184 | 0.086 |
|  | S_P_ | 1.177±0.370 | 1.005±0.281 | 0.138 |
|  | S_M_ | 1.413±0.300 | 1.452±0.348 | 0.651 |
|  | S_D_ | 1.469±0.342 | 1.497±0.362 | 0.763 |

**Supplementary Table S2. The best two identified features in each feature class in distinguishing K**i-67 status.

| Feature class | Feature name | AUC | ACC | Sensitivity | Specificity |
| --- | --- | --- | --- | --- | --- |
| S_I_ | Skewness | 0.589 (0.454-0.725) | 0.689 | 0.221 | 0.957 |
|  | Variance | 0.569 (0.422-0.716) | 0.703 | 0.370 | 0.894 |
| S_T_ | Kurtosis | 0.592 (0.456-0.728) | 0.622 | 0.404 | 0.852 |
|  | Skewness | 0.587 (0.446-0.728) | 0.675 | 0.556 | 0.745 |
| S_B_ | **Skewness** | **0.716 (0.587-0.845)** | **0.716** | **0.723** | **0.704** |
|  | Mean | 0.597 (0.456-0.736) | 0.621 | 0.489 | 0741 |
| S_P_ | **Skewness** | **0.647 (0.507-0.786)** | **0.648** | **0.851** | **0.444** |
|  | IQR | 0.576 (0.431-0.721) | 0.594 | 0.574 | 0.630 |
| S_M_ | **Max** | **0.612 (0.466-0.758)** | **0.702** | **0.787** | **0.556** |
|  | range | 0.592 (0.493-0.731) | 0.648 | 0.681 | 0.593 |
| S_D_ | Max | 0.609 (0.461-0.741) | 0.554 | 0.936 | 0.259 |
|  | Range | 0.518 (0.375-0.662) | 0.621 | 0.702 | 0.481 |
| Ratio between Areas | **S_P_/S_D_-mean** | **0.659** (0.527-0.792) | **0.662** | **0.511** | **0.815** |
|  | **S_P_/S_D_-IQR** | **0.657** (0.579-0.786) | **0.608** | **0.404** | **0.926** |
|  | S_T_/S_D_-kurtosis | 0.629 (0.496-0.763) | 0.635 | 0.532 | 0.778 |
|  | S_B_/S_M_-range | 0.627 (0.491-0.763) | 0.648 | 0.532 | 0.852 |
|  | S_P_/S_D_-mean | 0.576 (0.436-0.719) | 0.621 | 0.681 | 0.556 |

**Supplementary** Table S3. Best subset of features in breast tumour proximal regions

| Regions | Feature names |
| --- | --- |
| S_I_ | Max, variance, standard derivation, mean, range, skewness |
| S_T_ | Variance, range |
| S_B_ | Min, variance, standard derivation, mean, range, kurtosis |
| S_P_ | Variance, standard derivation, range, skewness |
| S_M_ | Max |
| S_D_ | Max |
| Statistical features | Min and range ADC value in S_I_; min and variance in S_T_; min value in S_B_; range, skewness and interquartile range in S_P_; kurtosis and skewness in S_M_. |
| Ratio features | S_I_/S_T_-IQR, S_I_/S_T_-kurtosis, S_I_/S_M_-max, S_T_/S_B_-min, S_T_/S_B_-IQR, S_T_/S_P_-kurtosis, S_B_/S_P_-IQR, S_B_/S_M_-skewness, S_B_/S_M_-standard derivation, S_P_/S_M_-variance, S_P_/S_D_-mean, S_P_/S_D_-min, S_M_/S_D_-IQR |
| All features | Min in S_B_, skewness in S_B_, IQR in S_P_, kurtosis in S_M_, skewness in S_P_, min and interquartile range between S_I_ and S_T_; skewness between S_I_ and S_P_; S_I_/S_M_-max, S_T_/S_B_-kurtosis, S_B_/S_P_-range, S_B_/S_M_-variance, S_P_/S_M_-variance, S_P_/S_M_-mean, S_M_/S_D_-IQR |

Note: S_I_: inner section of the tumour (-2 pixel); S_T_: whole tumour; S_B_: boundary region of the tumour, from -2 to 2 pixels; S_P_: peritumour stromal shell of 4 pixels outside the tumour; S_M_: intermediate stromal shell of 4 pixels outside the proximal stromal region; S_D_: distant stromal shell of 4 pixels outside S_M_. For example, S_I_/S_M_-max denotes the ratio of the max ADC value between S_I_ and S_M_.

**Supplementary Table S4. Percentage of selections in the** leave-one-out cross-validation test

| Feature | Selection frequency |
| --- | --- |
| S_P_ – skewness  S_I_ /S_T_ – IQR  S_B_ – skewness  S_I_ /S_T_ –min  S_B_ /S_M_ – variance  S_I_ /S_P_ - skewness  S_B_ - min | 70 (85%)  60(73%)  40(49%)  29(35%)  19(23%)  19(23%)  18(22%) |
| S_P_ - IQR | 16 (20%) |
| S_I_ /S_M_ - max | 11 (14%) |
| S_P_ /S_M_ - variance | 7 (9%) |
| S_T_ /S_D_ – kurtosis  S_B_ /S_P_ – range  S_M_ - kurtosis | 6 (8%)  6 (8%)  6 (7%) |
| S_M_ /S_D_ - IQR | 6 (7%) |
| S_P_ /S_M_ - mean | 4 (5%) |

Note: S_I_: inner section of the tumour (-2 pixel); S_T_: whole tumour; S_B_: boundary region of the tumour, from -2 to 2 pixels; S_P_: peritumour stromal shell of 4 pixels outside the tumour; S_M_: intermediate stromal shell of 4 pixels outside the proximal stromal region; S_D_: distant stromal shell of 4 pixels outside S_M_. For example, S_I_/S_M_-max denotes the ratio of the max ADC value between S_I_ and S_M_.

**Supplementary figures**

**
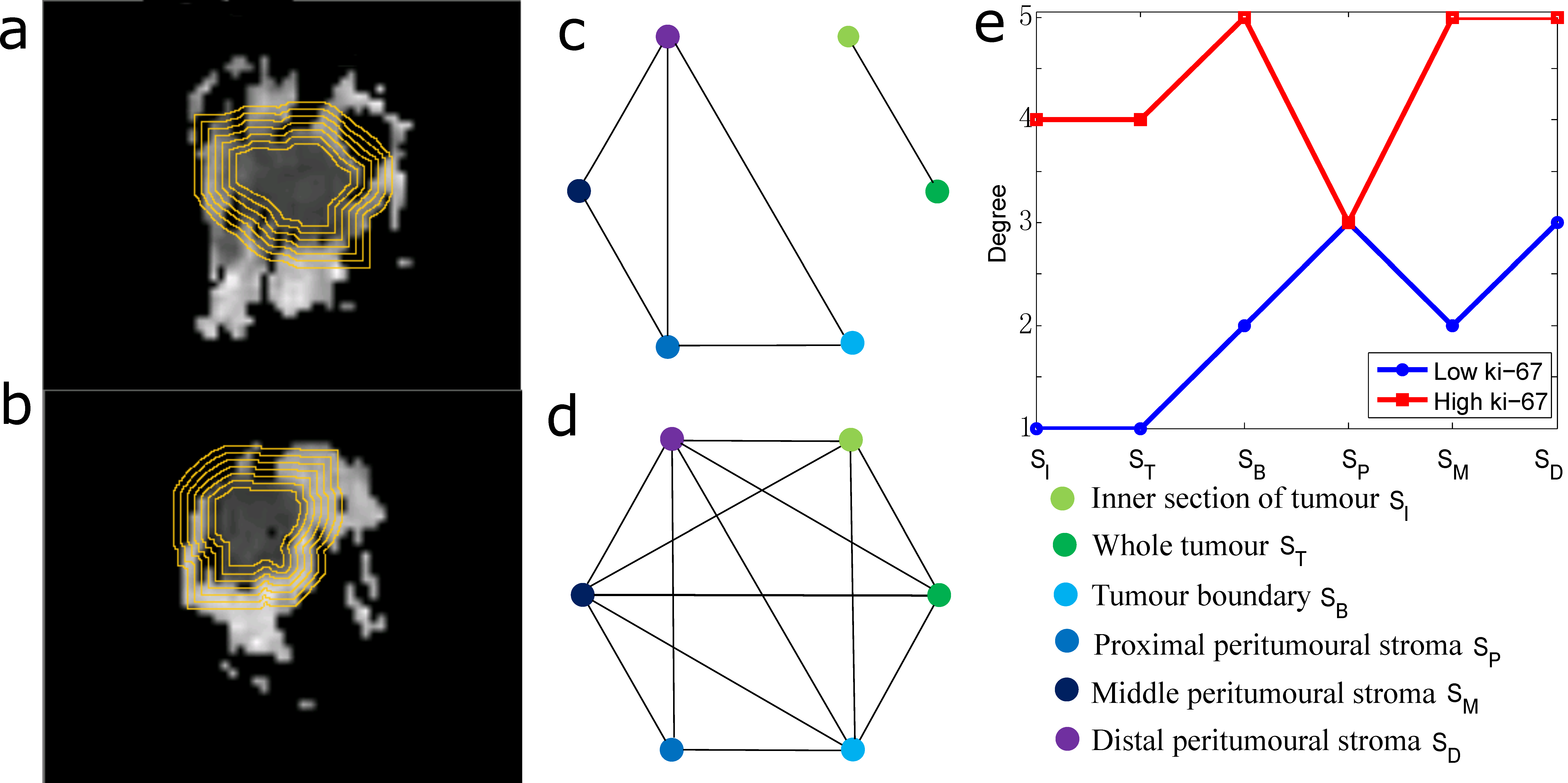
**

**Supplementary Figure S1. Representative example of ADC proximity map for low and high-Ki-67** **cases.** The fibroglandular proximity map was applied to an ADC image to calculate the proximal peritumoural stromal ADC map in a) low and b) high-Ki-67 cases. Graphs were established with nodes as tumour/stromal regions and edges as significant correlations of statistical features (i.e., max, min, mean, kurtosis and skewness of ADC values) between two regions (Bonferroni-corrected P value of Pearson's correlation coefficient less than 0.05) for the c) low-Ki-67 and d) high-Ki-67 breast tumours. The degree for each region in the Ki-67 samples is shown in e). Each node on the graph represents a tumour or peritumoural stroma, i.e., 1) inner section of a tumour, 2) whole tumour, 3) tumour boundary, 4) peritumoural stroma, 5) middle peritumoural stroma, and 6) distal peritumoural stroma.


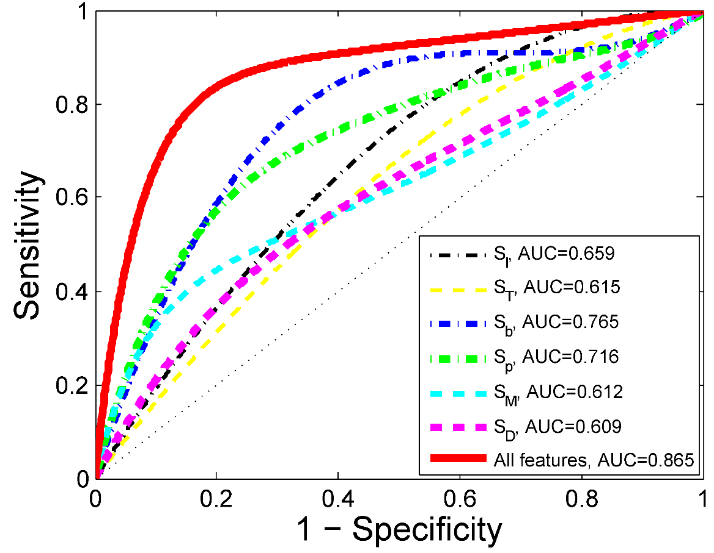


**Figure S2. Comparison of ROC curves for the prediction model in tumour and stromal regions**

**
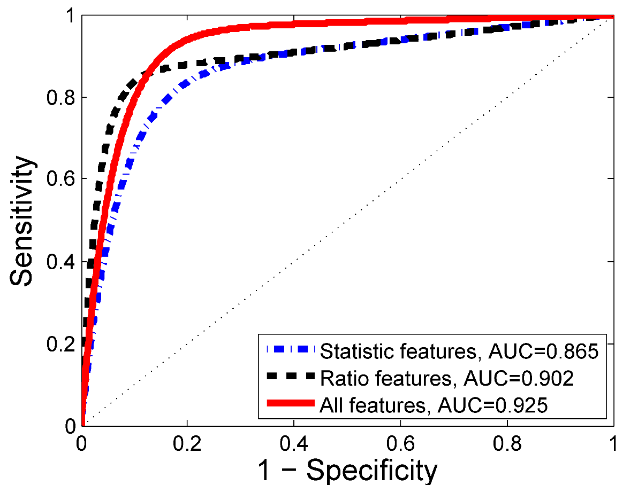
**

**Figure S3.** **Comparison of ROC curves for the prediction model using statistical features, ratio features and all features.**
